# Three-dimensional echocardiographic assessment of left ventricular geometric changes following acute myocardial infarction

**DOI:** 10.1007/s10554-022-02764-z

**Published:** 2022-12-06

**Authors:** Heba M. El-Naggar, Alaa S. Osman, Mohamed A. Ahmed, Amr A. Youssef, Tarek A. N. Ahmed

**Affiliations:** Department of Cardiovascular Medicine, Assiut University Heart Hospital, Assiut, 71526 Egypt

**Keywords:** Left ventricular remodeling, Myocardial infarction, 3D echocardiography, Global longitudinal strain

## Abstract

Acute ST-segment elevation myocardial infarction (STEMI) is associated with left ventricular (LV) structural and functional consequences. We aimed to elucidate LV geometric changes following STEMI using three-dimensional (3D) echocardiography (3DE) and to assess their functional implications using two-dimensional (2D) speckle tracking echocardiography (STE). The study included 71 patients with STEMI who underwent baseline and 6-month follow-up 2D- and 3DE. Measured parameters included LV dimensions, biplane volumes, wall motion assessment, 2D LV global longitudinal strain (GLS), and 3D LV volumes, sphericity index and systolic dyssynchrony index. According to 3DE, LV geometric changes were classified as, adverse remodeling, reverse remodeling, and minimal LV volumetric changes. The occurrence of in-hospital and follow-up major adverse cardiovascular events (MACE) was assessed among the study population. The incidence of developing adverse remodeling was 25.4% while that of reverse remodeling was 36.6%. Adverse remodeling patients had significantly higher in-hospital MACE. Reverse remodeling was associated with significantly improved GLS, that was less evident in those with minimal LV geometric changes, and non-significant improvement for adverse remodeling group. LV baseline 2D GLS significantly correlated with follow-up 3D volumes among both reverse and adverse remodeling groups. Female gender and higher absolute GLS change upon follow-up were significantly associated with reverse remodeling. ROC-derived cutoff for adverse remodeling reallocated a substantial number of patients from the minimal change group to the adverse remodeling. Following acute STEMI, two-dimensional GLS was associated with and potentially predictive of changes in LV volumes as detected by three-dimensional echocardiography.

## Introduction

In the setting of acute ST-segment-elevation myocardial infarction (STEMI), the injured myocardium may functionally recover or become irreversibly remodeled [1]. Adverse left ventricular (LV) remodeling is a dynamic process that starts early with the onset of myocardial ischemia, causing changes in myocardial geometry that may be followed by adverse cardiovascular events [2] and was shown to predict mortality [3]. Several treatments are now available to attenuate or partially reverse this phenomenon [4].

Transthoracic two-dimensional (2D) echocardiography has played an important role in identifying LV remodeling [5]. Three-dimensional (3D) echocardiography (3DE) provides a more precise analysis of LV morphology and function, that does not rely on geometric assumptions and is unaffected by foreshortening. Furthermore, 3DE showed comparable results to those provided by the current gold standard cardiac magnetic resonance (CMR) imaging, despite tendency of the former to underestimate volumes [6, 7]. Nevertheless, 3DE has the advantages of being more widely available and less time consuming and of less cost.

Myocardial deformation imaging, namely speckle tracking echocardiography (STE), has been shown to be an important load-independent tool for cardiac function analysis, compared to ejection fraction. 2D STE-derived strain values differentiate pathologically contracting segments from normal ones, assessing both regional and global myocardial function post myocardial infarction (MI) [8–10].

Accurate assessment of LV volumes together with functional assessment beyond the ejection fraction (EF) were postulated to be of clinical significance [11]. Determining those with LV adverse remodeling, those with reverse remodeling and those at the gray zone with minimal geometric changes, that may be prone to development of adverse remodeling, would impact the treatment offered.

Our study aimed to elucidate LV geometric changes following STEMI using 3DE and to assess their functional implications as determined with 2D STE. We hypothesize that 2D global longitudinal strain (GLS) might be associated/predictive of the 3DE-derived LV volumetric changes.

## Methods

This was a cross-sectional observational study conducted in the period from November 2018 to May 2019. The study included patients with the first episode of acute STEMI who presented to our institution, and who fulfilled baseline and 6-month follow-up echocardiographic studies. Patients underwent either primary percutaneous coronary intervention (PCI) or received thrombolytic therapy according to current guidelines for revascularization following STEMI [12]. Selection of the reperfusion strategy depended on institutional logistic and financial regulations, thus declining any potential clinically driven selection bias.

Patients with one or more of the following conditions were excluded; those with previous MI, PCI or coronary artery bypass graft (CABG), pre-existing significant valvular heart disease, pre-existing LV geometric changes with either hypertrophy or cardiomyopathy; as per the latest recommendations for chamber quantification guidelines [7], pre-existing significant arrhythmias providing difficulty upon 3DE analysis, those with poor image quality, and high-risk patients including; those presenting with hemodynamic instability necessitating mechanical ventilation or circulatory support. Patients developing heart failure (HF)/cardiogenic shock within the hospital stay after receiving reperfusion therapy were considered to have attained the study clinical endpoints of major adverse cardiac events (MACE) and thus were not excluded. Since defining the occurrence of LV geometric change would require baseline and follow-up echo, those who lost follow-up and those who died between the two echocardiographic time points were subsequently excluded from the analysis.

Patients undergoing primary PCI had immediate culprit vessel revascularization, with complete revascularization being staged either during the hospital stay or later after discharge. The use of either intracoronary or intravenous Glycoprotein IIb/IIIa inhibitors (GPIIb/IIIa) was left to the operators’ discretion. Patients receiving thrombolytic therapy were scheduled to undergo coronary angiography (CA) ± PCI shortly after hospital discharge for complete revascularization as necessitated. All patients routinely received dual antiplatelet therapy, statins, angiotensin-converting enzyme inhibitors (ACEIs)/angiotensin receptor blockers (ARBs) or beta-blockers in absence of respective contraindications.

All patients underwent baseline 2D- and 3DE within 24–72 h following the incident infarction and thereafter at a 6-month follow-up. Echocardiography was performed using Philips Epic7c ultrasound system (Philips Medical System/Andover/MA/USA), equipped with an S5-1 probe for 2D- and X5-1 probe for 3D-acquisition, respectively. Offline analysis for the 2D STE and 3D full volume recordings were done afterwards on a dedicated workstation using Philips Q-lab software/version 10.1. The observers performing the follow-up examinations were blinded to the baseline data.

Echocardiographic measures were performed according to the recent recommendations for chamber quantification guidelines [7] and included; 2D LV dimensions, biplane end-diastolic and end-systolic volumes, (EDV) and (ESV), biplane EF as well as the LV wall motion score index (WMSI). LV diastolic function parameters were also measured.

**Fig. 1:**
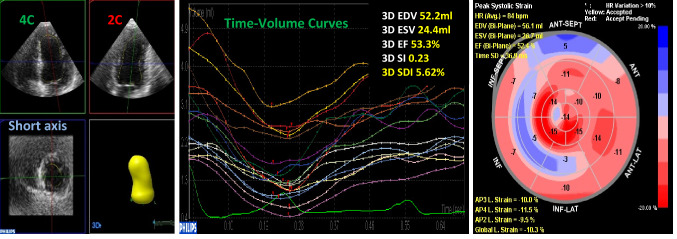
Example of the three-dimensional full-volume and two-dimensional speckle tracking derived measures, respectively: (left); 3D-derived 4-chamber, 2-chamber, and short-axis images, (middle); 3D-derived LV time-volume curves and calculated global volumes, ejection fraction, sphericity and dyssynchrony indices, (right); 2D-speckle tracking bull`s eye display of LV segmental and GLS

Two-D speckle tracking LV global longitudinal strain (GLS) was determined. ECG-triggered 2D gray-scale loops were acquired from the three standard apical imaging planes; apical 4-, 2- and 3-chamber views (A4C, A2C and A3C views) using a narrow sector and a frame rate of 60–90 frames/s. Offline analysis was done using the software automated cardiac motion quantification (aCMQ) feature. GLS was calculated as the average of the observed segmental values of the longitudinal peak systolic strain of all myocardial segments and displayed as a negative value measured in percentage (%), (Fig. 1).

Three-D echocardiographic assessments of LV volumes, sphericity index (SI) and systolic dyssynchrony index (SDI) were performed. A full volume data set was acquired from an A4C view in harmonic mode from four successive ECG-triggered heart beats during an end-expiratory breath-hold after adjusting for depth and focus. A dynamic pyramidal 3D data set was generated and stored. Offline analysis was performed using the software advanced cardiac 3D quantification (3DQA) feature. Three-dimensional LV EDV and ESV volumes were determined both at baseline and a 6-month follow-up, (Fig. 1). Accordingly, 3D LV EDV and ESV indices were calculated as the percentage change of 3D LV volumes at the 6-month follow-up compared to those at baseline.

Three-D LV SI signifies the ratio between the LV EDV and the volume which the LV would present had its shape been spherical. It was calculated by dividing the LV EDV by the volume of a sphere whose diameter is derived from the major end-diastolic LV long-axis; according to the expression: 3D LV SI = LVEDV/(4/3) π (D/2)^3^, (D is the major end-diastolic LV long-axis, measured as the longest distance between the center of the mitral annulus and the endocardial apex in the A4C view and identified by cropping the 3D dataset) [13]. Three-D SDI corresponds to the standard deviation of the LV 16-segment end-systolic contraction time corrected for the heart rate (RR interval) and displayed as percentage (%) values [14].

Definition of LV geometric changes was based on a 3D-volumetric percentage difference at 6-month follow-up compared to those at baseline [15]. Patients were classified into one of three groups: Adverse remodeling group; those who developed adverse remodeling defined as ≥ 15% increased LV EDV at follow-up compared to baseline [16], Reverse remodeling group; those who developed reverse remodeling defined as ≥ 15% decreased LV ESV at follow-up compared to baseline [17, 18] and Minimal-change group which included those not fitting in either reverse or adverse remodeling category.

Occurrence of in-hospital and follow-up adverse cardiac events was assessed among the study population. They included; fatal arrhythmia (ventricular tachycardia/fibrillation (VT/VF)), non-fatal arrhythmias (atrial fibrillation, frequent atrial/ventricular ectopics, accelerated idioventricular rhythm or ill-sustained VT), post-MI angina 24 h after incident infarction and up to 2 weeks, new-onset or recurrent unstable angina from 2 weeks following the incident MI, re-infarction, target lesion revascularization (TLR), and HF. Major adverse cardiac events (MACE) were defined as the cumulative occurrence of one or more of: re-infarction, TLR, HF, both in-hospital and up to 6-month follow-up. Death was not included in our study definition of MACE, as per exclusion criteria.

The study was approved by our institutional ethical committee, and patients consented to participate in the study.

## Statistical analysis

Data was analyzed using SPSS 20 statistical software (SPSS Inc./Chicago/IL/USA). Continuous data was expressed as mean ± SD or median (interquartile range) according to data distribution, while nominal data was expressed as frequency (percentage). Chi^2^-test was used to compare nominal data, while unpaired Student’s *t*-test was used to compare continuous variables. Paired Student’s *t*-test was used to compare echocardiographic data at 6-months follow-up with those at baseline. Non-parametric tests were used when appropriate. Pearson’s correlation coefficient (*r*) was used to determine the correlation between GLS and 3D LV volumes. Binary logistic regression analysis was performed for possible predictors of each of reverse and adverse remodeling among potentially relevant covariates. The diagnostic performance of the percentage change in LV 3D-EDV, EDV entailing adverse and reverse remodeling, respectively, and that for the follow-up 2D-GLS to predict cumulative MACE was assessed by receiver-operating characteristic (ROC) curve. The area under the curve (AUC) at 95% confidence interval (CI), as well as the optimal cut-off values, sensitivity, and specificity were reported. Reliability analysis was performed for intra- and inter-observer variability using intra-class correlation coefficient. Two-tailed *p*-value < 0.05 indicated statistical significance.

## Results

The current study initially recruited 87 patients with acute STEMI, out of whom, fifteen were lost to follow-up and one patient was reported to have died during the follow-up duration before having the 6-months echo performed. Final analysis included 71 patients for whom LV remodeling data was defined.

Based on 3D-quantification, 18 patients (25.4%) had adverse remodeling, 26(36.6%) had reverse remodeling and 27(38.0%) had minimal positive or negative LV volumetric changes. Demographic data and clinical outcomes among the study groups were demonstrated in Table 1. Majority of patients among the three groups were males, with significantly higher proportion of females among those having LV reverse remodeling compared to the other two groups. Total ischemic time, Killip class, the proportion of those with anterior infarction and the reperfusion therapy adopted were comparable among the study groups, similarly were the levels of cardiac enzymes and degree of ST-segment resolution. Significantly higher use of BB was seen among both the reverse remodeling and the minimal-change groups. Complete revascularization was attained in 80.3% of our patient population with comparable proportion among the three groups.

**Table 1:** Demographic data and clinical outcomes among the study groups

	Minimal LV geometric changes (n = 27) (38.0%)	Adverse remodeling (n = 18) (25.4%)	Reverse remodeling (n = 26) (36.6%)	*P* value
Age (years)	56.81 ± 9.72	55.44 ± 9.25	55.92 ± 9.77	0.89
Male gender	26 (96.3%)	17 (94.4%)	19 (73.1%)	0.02
Smoking	22 (81.5%)	16 (88.9%)	17 (65.4%)	0.15
Hypertension	7 (25.9%)	1 (5.6%)	5 (19.2%)	0.22
Diabetes mellitus	5 (18.5%)	1 (5.6%)	8 (30.8%)	0.11
Family history of CAD	1 (3.7%)	1 (5.6%)	3 (11.5%)	0.51
BMI (kg/m^2^)	26.33 ± 4.36	25.33 ± 4.54	28.44 ± 6.10	0.12
Clinical, ECG and lab data				
Killip class III–IV	1 (3.7%)	1 (5.6%)	3 (11.5%)	0.51
Total ischemic time (min)	319.44 ± 171.83	322.78 ± 163.5	398.46 ± 178.87	0.19
Type of MI				
Anterior MI	17 (63.0%)	10 (55.6%)	16 (61.5%)	0.87
Non-anterior MI	10 (37.0%)	8 (44.4%)	10 (38.5%)	
ST-segment resolution				
No resolution	5 (18.5%)	2 (11.1%)	4 (15.4%)	0.75
Partial resolution	12 (44.4%)	11 (61.1%)	11 (42.3%)	
Complete resolution	10 (37.0%)	5 (27.8%)	11 (42.3%)	
Cardiac enzymes				
Peak CK (U/L)	2130 (1460–3070)	1882 (889–2773)	1334 (784–2595)	0.46
Peak CK-MB (U/L)	200 (117–316)	252 (119–465)	244 (114–351)	0.66
Admission cTpI (ng/ml)	3.5 (0.3–10.3)	3.1 (0.4–45.3)	2.5 (1.0–38.4)	0.86
Reperfusion therapy				
PPCI (%)	14 (51.9%)	11 (61.1%)	17 (65.4%)	0.59
TT (%)	13 (48.1%)	7 (38.9%)	9 (34.6%)	
Complete revascularization	19 (73.1%)	17 (94.4%)	21 (84.0%)	0.18
Medications				
GPIIb/IIIa inhibitors	6 (22.2%)	7 (38.9%)	7 (28.0%)	0.47
ACEIs/ARBs	25 (92.6%)	15 (83.3%)	24 (92.3%)	0.53
B-Blockers	24 (88.9%)	10 (55.6%)	23 (88.5%)	0.009
In-hospital duration (days)	2.93 ± 0.61	3.33 ± 0.97	3.19 ± 0.84	0.22
In-hospital Events				
VT/VF	1 (3.7%)	0 (0.0%)	2 (7.7%)	0.45
Nonfatal arrhythmias	1 (3.7%)	1 (5.6%)	1 (3.8%)	0.94
Post-MI angina	1 (3.7%)	0 (0.0%)	0 (0.0%)	0.43
Reinfarction	0 (0.0%)	1 (5.6%)	1 (3.8%)	0.52
TLR	0 (0.0%)	0 (0.0%)	0 (0.0%)	NA
HF	2 (7.4%)	7 (38.9%)	1 (3.8%)	0.002
Followup cumulative events				
VT/VF	1 (3.7%)	0 (0.0%)	2 (7.7%)	0.45
Nonfatal arrhythmias	1 (3.7%)	1 (5.6%)	1 (3.8%)	0.94
Unstable angina	4 (14.8%)	1 (5.6%)	0 (0.0%)	0.10
Reinfarction	0 (0.0%)	1 (5.6%)	1 (3.8%)	0.50
TLR	0 (0.0%)	0 (0.0%)	0 (0.0%)	NA
HF	2 (7.4%)	7 (38.9%)	1 (3.8%)	0.002
In-hospital MACE	2 (7.4%)	8 (44.4%)	2 (7.7%)	0.001
Follow-up Cumulative MACE	2 (7.4%)	8 (44.4%)	2 (7.7%)	0.001

*ACEIs/ARBs* angiotensin-converting enzyme inhibitors/angiotensin receptor blockers, *BMI* body mass index, *CAD* coronary artery disease, *CK* creatine kinase, *CK-MB* creatine kinase-myoglobin fraction, *cTpI* cardiac troponin-I, *ECG* electrocardiography, *HF* heart failure, *MACE* major adverse cardiovascular events, *MI* myocardial infarction, *NA* not applicable, *PPCI* primary percutaneous coronary intervention, *TLR* target lesion revascularization, *TT* thrombolytic therapy, *VT/VF* ventricular tachycardia/fibrillation

The cumulative MACE was significantly higher among patients with adverse remodeling, mainly attributed to HF occurring during the in-hospital course with no further events occurring at follow-up. Otherwise, there was no difference in the rates of other events, with no TLR events being recorded either in-hospital or at follow-up (Table 1).

**Fig. 2:**
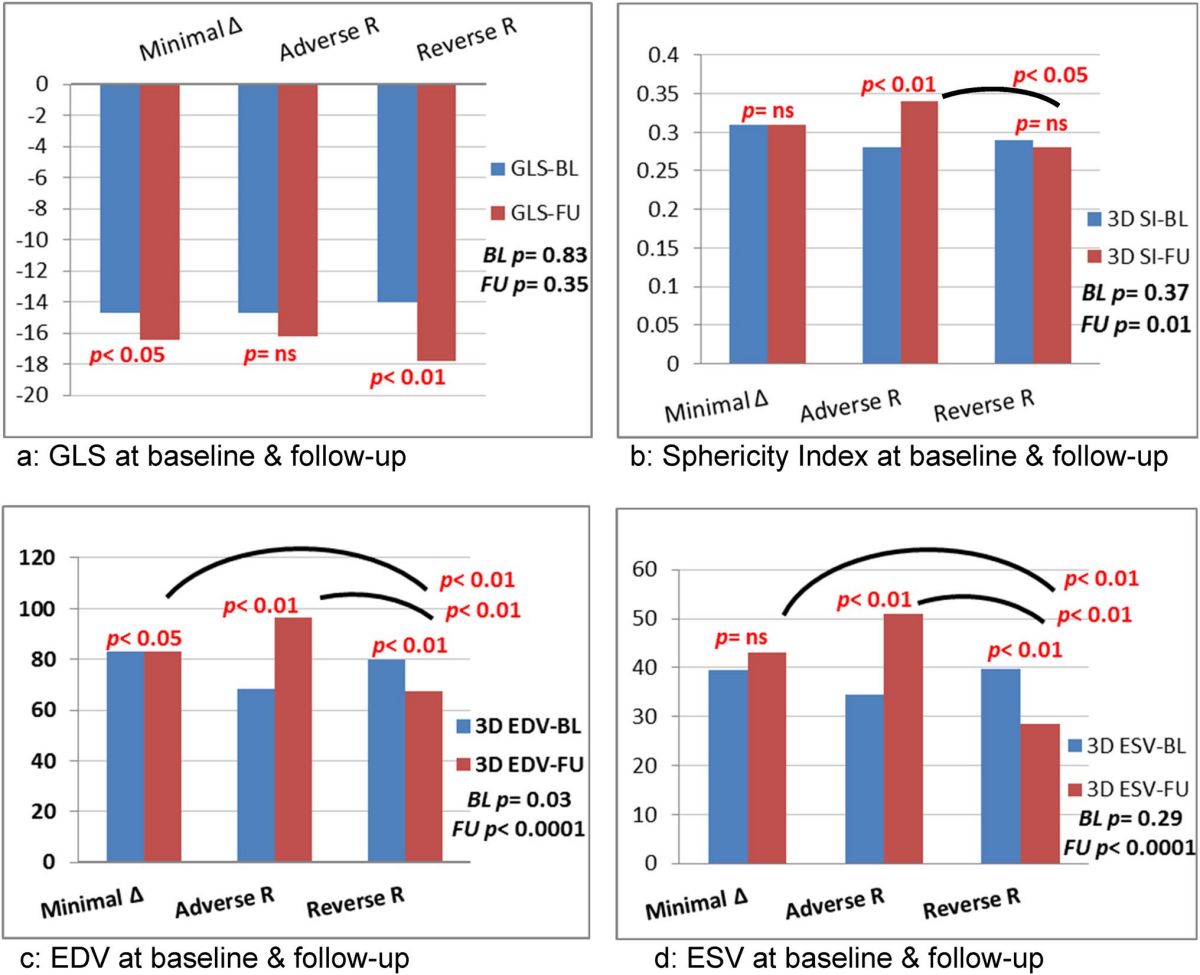
Left ventricular changes among different remodeling groups. **a** Global longitudinal strain, **b** Sphericity index, **c** End-diastolic volume, **d** End-systolic volume. Parameters were displayed at baseline and six-month follow-up with paired analysis within each group and one-way ANOVA between groups. *3D* three-dimensional, *Adverse R* adverse remodeling, *BL* baseline, *EDV* end-diastolic volume, *ESV* end-systolic volume, *EF* ejection fraction, *FU* follow-up, *GLS* global longitudinal strain, *Minimal Δ* minimal change, *Reverse R* reverse remodeling, *SI* sphericity index. Within-group paired analysis of echocardiographic data at 6-month follow-up compared to that at baseline is indicated by p-value displayed above each two corresponding bars. One-way ANOVA comparing echocardiographic data between different LV remodeling groups is indicated as Baseline (BL) p-value and Follow-up (FU) p-value. Post-hoc Bonferroni analysis is indicated by the black arcs within the graph and their corresponding p-value. **a** GLS at baseline & follow-up, **b** Sphericity index at baseline & follow-up, **c** EDV at baseline & follow-up and **d** ESV at baseline & follow-up

Exploring the 2D echocardiographic data among the different groups showed no significant difference regarding baseline LV dimensions, biplane volumes, EF, or diastolic function parameters, similarly were the baseline WMSI and GLS values. On follow-up, significantly larger biplane EDV and ESV volumes with lower EF were noted among the adverse remodeling group. Follow-up WMSI and GLS were comparable among the three groups. However, on paired analysis, significant improvement of GLS at follow-up compared to baseline was evident among the reverse remodeling group (p < 0.01) and to less extent among the minimal-change group (p < 0.05), while non-significant difference in GLS among adverse remodeling patients (Table 2 and Fig. 2A). The absolute GLS change from baseline to follow-up was calculated and showed significant difference between the three groups (p = 0.04), with the highest improvement among the reverse remodeling group (Table 2).

**Table 2:** Two-dimensional echocardiographic data among the study groups

	Minimal LV geometric changes (n = 27) (38.0%)	Adverse remodeling (n = 18) (25.4%)	Reverse remodeling (n = 26) (36.6%)	*P* value between groups
2D Echo data				
Baseline				
LV EDD (mm)	5.27 ± 0.48	5.11 ± 0.50	5.17 ± 0.67	0.63
LV ESD (mm)	3.59 ± 0.64	3.50 ± 0.65	3.52 ± 0.67	0.88
RWT	0.35 ± 0.06	0.37 ± 0.06	0.37 ± 0.08	0.54
LV mass index	97.81 ± 19.24	102.39 ± 24.19	99.54 ± 26.53	0.81
LV EDV (ml)	91.23 ± 23.37	83.93 ± 21.03	82.30 ± 23.20	0.32
LV ESV (ml)	45.10 ± 19.42	41.92 ± 17.24	41.17 ± 15.37	0.69
LV EF (%)	51.80 ± 9.94	51.02 ± 9.78	50.98 ± 9.61	0.94
WMSI	1.53 ± 0.27	1.57 ± 0.27	1.49 ± 0.26	0.57
Follow-up				
LV EDD (mm)	5.62 ± 0.65**	5.53 ± 0.76*	5.39 ± 0.75	0.53
LV ESD (mm)	3.83 ± 0.86	3.88 ± 0.75	3.59 ± 0.66	0.38
RWT	0.31 ± 0.05*	0.34 ± 0.06	0.34 ± 0.06	0.29
LV mass index	102.78 ± 24.35	106.22 ± 30.55	96.15 ± 27.03	0.44
LV EDV (ml) (c)	102.11 ± 35.77*	114.71 ± 37.70**	83.14 ± 20.04	0.005
LV ESV (ml) (b, c)	51.66 ± 33.05	55.74 ± 24.02*	34.45 ± 15.16*	0.01
LV EF (%)	52.74 ± 8.72	52.18 ± 8.72	59.56 ± 10.05**	0.03
WMSI	1.40 ± 0.28**	1.44 ± 0.29**	1.31 ± 0.18**	0.19
Doppler and TDI data				
Baseline				
E/A ratio	1.04 ± 0.35	0.87 ± 0.39	0.91 ± 0.35	0.28
Deceleration time (msec)	151.30 ± 49.07	149.56 ± 48.76	151.31 ± 47.35	0.99
IVRT (msec)	78.00 ± 13.34	80.89 ± 18.29	79.62 ± 17.15	0.83
E/e´(average)	9.22 ± 2.55	8.68 ± 3.51	8.97 ± 2.59	0.81
Follow-up				
E/A ratio	1.14 ± 0.82	1.12 ± 0.61*	0.91 ± 0.36	0.35
Deceleration time (msec)	175.52 ± 40.82	160.17 ± 33.12	171.88 ± 36.02*	0.39
IVRT (msec)	90.07 ± 12.31**	87.33 ± 16.82	85.23 ± 16.10	0.5
E/e´(average)	8.67 ± 4.83	9.41 ± 5.02	8.60 ± 2.24	0.79
2D Speckle tracking				
Baseline				
GLS (%)	− 14.67 ± 4.18	− 14.69 ± 5.08	− 14.05 ± 3.62	0.83
4ch-LS (%)	− 14.74 ± 4.57	− 14.19 ± 5.11	− 14.45 ± 4.16	0.92
2ch-LS (%)	− 15.09 ± 4.87	− 15.14 ± 5.69	− 13.60 ± 3.91	0.44
3ch-LS (%)	− 14.19 ± 3.81	− 14.83 ± 5.47	− 14.06 ± 4.11	0.83
Follow-up				
GLS (%)	− 16.45 ± 3.89*	− 16.19 ± 3.95	− 17.75 ± 4.01**	0.35
4ch-LS (%)	− 17.04 ± 4.82*	− 16.04 ± 4.16	− 18.36 ± 4.21**	0.22
2ch-LS (%)	− 16.23 ± 4.02	− 16.51 ± 3.88	− 17.88 ± 4.41**	0.31
3ch-LS (%)	− 16.10 ± 3.47**	− 16.05 ± 4.53	− 16.91 ± 4.15**	0.68
Absolute GLS change (%)	1.87 ± 3.42	1.5 ± 3.15	3.74 ± 3.27	**0.04**

Absolute GLS change (%) = Absolute follow-up GLS—Absolute baseline GLS

Paired analysis of echocardiographic data at 6-month follow-up compared to that at baseline: *denotes *p*-value < 0.05 and **denotes *p*-value < 0.01

One-way ANOVA and Post-hoc Bonferroni test comparing echocardiographic data between and within different LV remodeling groups: (a) denotes *p*-value < 0.05 between the group with minimal LV geometric changes and that with adverse remodeling, (b) denotes *p*-value < 0.05 between the group with minimal LV geometric changes and that with reverse remodeling and (c) denotes *p*-value < 0.05 between the group with adverse remodeling and that with reverse remodeling

*2D* two-dimensional, *2ch* two-chamber, *3ch* three-chamber, *4ch* four-chamber, *EDD* end-diastolic diameter, *ESD* end-systolic diameter, *EDV* end-diastolic volume, *ESV* end-systolic volume, *EF* ejection fraction, *IVRT* isovolumic relaxation time, *LV* left ventricle, *LS* longitudinal strain, *GLS* global longitudinal strain, *TDI* tissue Doppler imaging, *WMSI* wall motion score index

Regarding the 3D-derived parameters, Table 3 showed the changes marking the three different groups. Follow-up volumes showed significant difference between the three groups with the post-hoc analysis showing significantly smaller systolic and diastolic volumes among the reverse remodeling compared to the other two groups, while non-significant difference between the minimal LV geometric change group and the adverse remodeling group. Figure 2C, D demonstrated the LV 3D-volumetric changes among the different study groups at baseline and follow-up.

**Table 3:** Three-dimensional echocardiographic data among the study groups

	Minimal LV geometric changes (n = 27) (38.0%)	Adverse remodeling (n = 18) (25.4%)	Reverse remodeling (n = 26) (36.6%)	*P* value between groups
3D Echo data				
Baseline				
LV EDV (ml) (a)	83.28 ± 24.70	68.17 ± 12.99	79.72 ± 15.92	0.03
LV ESV (ml)	39.56 ± 15.01	34.50 ± 10.26	39.84 ± 9.72	0.29
LV EF (%)	52.70 ± 7.56	49.33 ± 8.59	50.31 ± 6.88	0.3
LV SI	0.31 ± 0.09	0.28 ± 0.07	0.29 ± 0.06	0.37
LV SDI (%)	6.98 ± 4.59	8.85 ± 4.55	6.60 ± 4.70	0.26
Follow-up				
LV EDV (ml) (b, c)	82.96 ± 21.25*	96.59 ± 19.06**	67.44 ± 16.27**	< 0.0001
LV ESV (ml) (b,c)	43.01 ± 16.99	50.91 ± 15.04**	28.41 ± 8.10**	< 0.0001
LV EF (%) (b, c)	49.58 ± 7.81	47.32 ± 8.10	57.45 ± 7.41**	< 0.0001
LV SI (c)	0.31 ± 0.08	0.34 ± 0.08**	0.28 ± 0.05	0.01
LV SDI (%)	6.91 ± 3.89	7.76 ± 5.42	5.01 ± 3.42	0.08
Remodeling data				
EDV Index (%) (a, b, c)	6.7 (− 18.0–12.5)	44.8 (23.4–58.0)	− 11.9 (− 28.3–3.4)	< 0.0001
ESV Index (%) (a, b, c)	6.0 (− 6.5–21.2)	39.3 (23.2–72.0)	− 28.4 (− 35.5–19.6)	< 0.0001

Paired analysis of echocardiographic data at 6-month follow-up compared to that at baseline: *denotes *p*-value < 0.05 and **denotes *p*-value < 0.01

One-way ANOVA and Post-hoc Bonferroni test comparing echocardiographic data between and within different LV remodeling groups: (a) denotes *p*-value < 0.05 between the group with minimal LV geometric changes and that with adverse remodeling, (b) denotes *p*-value < 0.05 between the group with minimal LV geometric changes and that with reverse remodeling and (c) denotes *p*-value < 0.05 between the group with adverse remodeling and that with reverse remodeling

*3D* three-dimensional, *EDV* end-diastolic volume, *ESV* end-systolic volume, *EF* ejection fraction, *LV* left ventricle, *SDI* systolic dyssynchrony index, *SI* sphericity index

Three-D derived SI showed remarkably lower values among the reverse remodeling group compared to the adverse remodeling one at follow-up. The latter demonstrated significant increase in the 3D-SI at follow-up compared to baseline values on paired analysis (Table 3 and Fig. 2B). There was a statistically non-significant difference in the SDI values between or within the three groups both at baseline and follow-up (Table 3).

Studying the above echocardiographic parameter among those who developed MACE versus those who did not (Table 4), showed significantly larger LV volumes with significantly reduced GLS values at follow-up among those developing MACE.

**Table 4:** Echocardiographic data according to the occurrence of MACE

	No MACE (n = 59) (83.1%)	MACE (n = 12) (16.9%)	*P* value
Baseline 2D echo data			
LV EDD (mm)	5.21 ± 0.60	5.11 ± 0.33	0.54
LV ESD (mm)	3.59 ± 0.65	3.29 ± 0.60	0.14
RWT	0.36 ± 0.07	0.38 ± 0.06	0.39
LV mass index	99.36 ± 24.36	100.83 ± 16.45	0.84
LV EDV (ml)	85.05 ± 22.66	91.28 ± 23.70	0.39
LV ESV (ml)	42.25 ± 16.86	45.82 ± 19.93	0.51
LV EF (%)	51.36 ± 9.48	51.02 ± 10.89	0.91
WMSI	1.49 ± 0.25	1.70 ± 0.25	0.01
Follow-up 2D echo data			
LV EDD (mm)	5.47 ± 0.73	5.73 ± 0.61	0.26
LV ESD (mm)	3.71 ± 0.78	3.99 ± 0.67	0.25
RWT	0.33 ± 0.06	0.32 ± 0.05	0.73
LV mass index	99.51 ± 26.09	109.67 ± 30.51	0.23
LV EDV (ml)	93.10 ± 30.17	124.21 ± 38.43	0.003
LV ESV (ml)	43.52 ± 26.10	60.55 ± 26.26	0.04
LV EF (%)	55.58 ± 11.51	52.75 ± 8.48	0.42
WMSI	1.35 ± 0.25	1.51 ± 0.23	0.04
Baseline Doppler and TDI data			
E/A ratio	0.94 ± 0.36	0.99 ± 0.38	0.69
Deceleration time (msec)	151.17 ± 47.79	149.33 ± 49.18	0.9
IVRT (msec)	79.86 ± 16.31	76.67 ± 14.22	0.53
E/e´(average)	8.64 ± 2.37	10.72 ± 4.05	0.01
Follow-up Doppler and TDI data			
E/A ratio	1.04 ± 0.62	1.08 ± 0.73	0.86
Deceleration time (msec)	169.54 ± 37.98	174.00 ± 34.65	0.7
IVRT (msec)	86.12 ± 13.37	94.92 ± 20.02	0.06
E/e´(average)	8.53 ± 3.58	10.33 ± 5.97	0.16
Baseline 2D-STE data			
GLS (%)	− 14.85 ± 4.15	− 12.50 ± 3.96	0.07
4ch-LS (%)	− 14.89 ± 4.56	− 12.55 ± 3.83	0.1
2ch-LS (%)	− 14.96 ± 4.79	− 12.55 ± 4.29	0.11
3ch-LS (%)	− 14.70 ± 4.24	− 12.39 ± 4.46	0.09
Follow-up 2D-STE data			
GLS (%)	− 17.44 ± 3.87	− 14.00 ± 3.14	0.005
4ch-LS (%)	− 17.92 ± 4.39	− 14.06 ± 3.49	0.006
2ch-LS (%)	− 17.37 ± 4.11	− 14.60 ± 3.63	0.03
3ch-LS (%)	− 17.03 ± 3.85	− 13.25 ± 3.04	0.002
Baseline 3D Echo data			
LV EDV (ml)	78.46 ± 20.98	76.59 ± 13.67	0.76
LV ESV (ml)	38.41 ± 12.56	38.22 ± 10.55	0.96
LV EF (%)	51.11 ± 7.31	50.27 ± 9.34	0.73
LV SI	0.30 ± 0.08	0.29 ± 0.07	0.7
LV SDI (%)	7.07 ± 4.80	8.55 ± 3.71	0.31
Follow-up 3D echo data			
LV EDV (ml)	76.73 ± 20.14	100.41 ± 20.63	< 0.0001
LV ESV (ml)	36.90 ± 14.84	53.24 ± 17.65	0.001
LV EF (%)	52.67 ± 8.61	48.06 ± 8.97	0.09
LV SI	0.30 ± 0.07	0.33 ± 0.09	0.24
LV SDI (%)	6.06 ± 3.77	8.24 ± 6.07	0.1

*2D* two-dimensional, *2ch* two-chamber, *3D* three-dimensional, *3ch* three-chamber, *4ch* four-chamber, *EDD* end-diastolic diameter, *ESD* end-systolic diameter, *EDV* end-diastolic volume, *ESV* end-systolic volume, *EF* ejection fraction, *IVRT* isovolumic relaxation time, *LV* left ventricle, *LS* longitudinal strain, *GLS* global longitudinal strain, *RWT* relative wall thickness, *TDI* tissue Doppler imaging, *WMSI* wall motion score index, *SDI* systolic dyssynchrony index, *SI* sphericity index

**Fig. 3:**
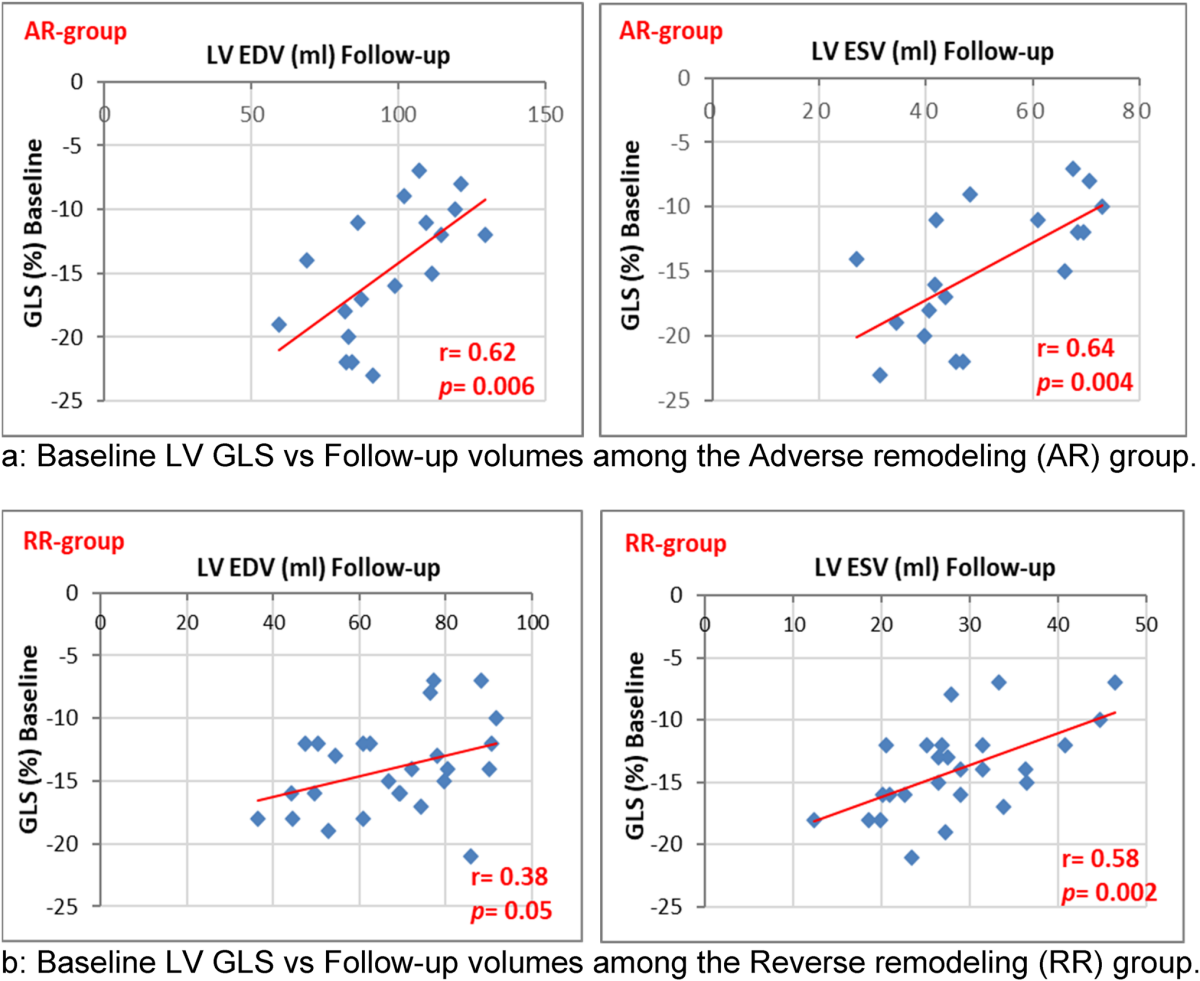
Correlation between baseline left ventricular global longitudinal strain and follow-up LV end-diastolic and end-systolic volumes among the Adverse remodeling group **a** and Reverse remodeling group **b**. *AR* adverse remodeling, *EDV* end-diastolic volume, *ESV* end-systolic volume, *GLS* global longitudinal strain, *RR* reverse remodeling, *r* (correlation coefficient) and *p* (significance of correlation). **a** Baseline LV GLS vs Follow-up volumes among the Adverse remodeling (AR) group and **b** Baseline LV GLS vs Follow-up volumes among the Reverse remodeling (RR) group

Exploring the association between functional and structural LV parameters showed significant correlation between baseline 2D GLS and follow-up 3D volumes among each of the adverse and reverse remodeling groups (Fig. 3).

Regression analysis was performed for possible predictors of each of reverse and adverse remodeling (Table 5). Although statistically non-significant, male gender had higher odds of having adverse remodeling. On the other hand, females had significantly higher odds of having reverse remodeling. The use of B-blockers significantly lowered the odds of developing adverse remodeling. Higher absolute GLS change upon follow-up was significantly associated with reverse remodeling, while baseline LV EDV was a significant predictor for adverse remodeling.

**Table 5:** Predictors of Reverse and Adverse remodeling

	Univariate analysis for predictors of Reverse Remodeling			Univariate analysis for predictors of Adverse Remodeling		
Variables	OR	95% CI	*P* value	OR	95% CI	*P* value
Age (years)	0.99	0.94–1.04	0.88	0.99	0.93–1.05	0.72
Gender (male)	0.12	0.02–0.66	0.01	3.02	0.35–26.00	0.31
B-Blockers	2.48	0.62–9.87	0.19	0.16	0.04–0.56	0.004
WMSI	0.43	0.06–2.70	0.36	2.57	0.34–19.22	0.35
2D baseline GLS (%)	1.03	0.92–1.16	0.54	0.98	0.86–1.11	0.77
Absolute GLS change (%)	1.21	1.02–1.43	0.02	0.88	0.74–1.05	0.16
3D LV EDV baseline (ml)	1.01	0.98–1.03	0.61	0.95	0.92–0.99	0.01
3D LV ESV baseline (ml)	1.01	0.97–1.05	0.44	0.95	0.90–1.01	0.11

Absolute GLS change (%) = Absolute follow-up GLS—Absolute baseline GLS

*2D* two-dimensional, *3D* three-dimensional, *CI* confidence interval, *EDV* end-diastolic volume, *ESV* end-systolic volume, *GLS* global longitudinal strain, *LV* left ventricle, *OR* odd’s ratio, *WMSI* wall motion score index

**Fig. 4:**
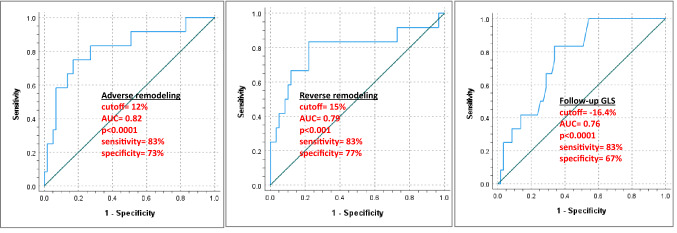
Receiver-operating characteristic (ROC) analysis of the percentage change in 3D end-diastolic volume (Adverse remodeling) (left), the percentage change in in 3D end-systolic volume (Reverse remodeling) (middle), and the percentage of 2D global longitudinal strain at follow-up (right) for the cumulative MACE. *AUC* area under the curve, *GLS* global longitudinal strain, *MACE* major adverse cardiovascular events

Receiver-operating analysis identified a cutoff of 15% change in 3D-ESV (AUC = 0.79, 95%CI (0.61–0.96), p < 0.001, sensitivity = 83% and specificity = 77%), 12% change in 3D-EDV (AUC = 0.82, 95%CI (0.67–0.96), p < 0.0001, sensitivity = 83% and specificity = 73%), and − 16.4% for follow-up 2D-GLS (AUC = 0.76, 95%CI (0.64–0.89), p < 0.0001, sensitivity = 83% and specificity = 67%) to be associated with cumulative MACE (Fig. 4). Based on our analysis, the newly derived ROC cutoff for adverse remodeling resulted in reallocation of 8 patients from the minimal geometric change group into the adverse remodeling one.

Reproducibility analysis testing for intra- and inter-observer variability was performed for a random sample of thirty-eight baseline and follow-up measures of 2D-GLS and 3D-derived LV volumes. The intraclass correlation coefficient of intra-observer variability for GLS, LV EDV and ESV were 0.94, 0.92, and 0.91, respectively (p < 0.0001 for all). The intraclass correlation coefficient of inter-observer variability for GLS, LV EDV and ESV were 0.93, 0.89, and 0.90, respectively (p < 0.0001 for all).

## Discussion

Acute myocardial infarction is associated with LV structural and functional alterations which are reflected on the clinical outcomes. We thought to look more in-depth studying LV geometric changes following MI, based on 3D LV volumetric assessment. Hence, we classified LV remodeling patterns into three groups, adverse remodeling, reverse remodeling, and minimal LV volumetric change (subtle positive or negative geometric changes). We also tried to explore their associated LV functional changes beyond the EF, using 2D GLS.

Previous studies demonstrated adverse remodeling versus no adverse remodeling following STEMI [16]. Now, in the era of primary PCI with the possibility of attaining immediate revascularization of the infarct-related artery, it has been increasingly noticed that reverse remodeling stands as an important bright-side counterpart among the LV geometric changes post-STEMI [19]. Previous studies tackled the concept of reverse remodeling in the course of HF management with either pharmacological (namely, ACEIs and/or B-blockers) [20, 21], or non-pharmacological measures (namely, cardiac resynchronization therapy) [18].

The reported incidence of adverse and reverse remodeling in the setting of acute MI following reperfusion therapy was 30–35% [10] and 40% [22], respectively. Results of our study showed that the overall incidence of adverse remodeling was 25.4% and that of reverse remodeling was 36.6%. Moreover, our study reported on the incidence of a rather overlooked group with minimal LV geometric changes, which was 38%. Reverse remodeling was associated with significantly improved GLS at follow-up. Our study also demonstrated a significant moderate correlation between baseline LV GLS and both baseline and follow-up 3D volumes among the adverse and reverse remodeling groups.

Our results showed higher propensity of females to develop reverse remodeling. This was concordant with previous studies and systematic reviews [23–25], which might be related to gender-specific variations in response to either the index cardiac event or the subsequently implemented therapies.

In our study, we used 3D echo-derived LV volumes based on their higher ability to accurately define adverse and reverse LV remodeling considering the 3D LV structure rather than only single or biplane assessment liable to image-plane positioning errors and geometric assumptions, particularly in the setting of distorted LV shape following an infarction. Three-D LV volumes have been shown to be up to three times more accurate than 2D volumes [26] and as accurate as the gold standard CMR-derived ones [6, 27].

Incidence of in-hospital MACE and hence follow-up cumulative MACE was significantly higher among the adverse remodeling group compared to the others, driven by the higher percentage of patients developing in-hospital HF. Consequently, with more in-hospital HF, there was less use of B-blockers among our adverse remodeling group. B-blockers are known to hinder LV adverse remodeling changes [4]. Upon follow-up, patients among the MACE group developed significantly larger LV volumes with significantly reduced GLS values.

Previous studies demonstrated that adverse remodeling was associated with increased incidence of HF [2, 28] as well as overall MACE [29]. In a prospective cohort of 285 patients with STEMI who underwent revascularization, developing adverse LV remodeling at 6 months, defined as ≥ 12% increase in both LVESV and LVEDV by CMR, was associated with higher 5-year composite of all-cause death and HF hospitalization [30].

Our study showed proximity of follow-up 3D volumes between the minimal change group and the adverse remodeling group. Hence, the former group represents a considerable proportion of patients that might be at the verge of progressing to adverse remodeling. Those need to be looked at carefully as targets for prevention of HF where cardiac protective therapies balance the detrimental effects of ongoing cardiac insult. Early initiation and maintenance of known anti-remodeling drugs (Beta-blockers, ACEIs/ARBs, and mineralocorticoid receptor antagonists) or the newly introduced sacubitril/valsartan and sodium–glucose cotransporter two inhibitor (SGLT2i) would benefit these patients. A study of long-term ventricular remodeling after revascularization for STEMI as assessed by CMR, showed that LV remodeling, whether adverse or reverse, is an ongoing process continuing at least up to 2 years following the acute insult, involving both the infarct zone and remote [31].

Speckle-tracking strain imaging has the advantage of being load-independent, it provides information on myocardial tissue function, allowing better differentiation between passive and active LV-segmental motion. Moreover, being a semiautomatic method, it provides a more objective interpretation of LV systolic function [32]. Pair-wise analysis within the remodeling groups showed significant improvement in GLS values among those with reverse remodeling. Furthermore, there was a significant moderate correlation between baseline GLS and follow-up 3D LV volumes among each of the reverse or adverse remodeling groups, particularly for ESV. As per our analysis, improvement in 2D-GLS was a potential predictor for reverse remodeling.

This highlights that reverse remodeling, denoted by improved LV volumes namely ESV, was not only associated with improved EF but was better reflected by subclinical functional improvement as demonstrated using the 2D-GLS. The use of advanced 3D-GLS together with the 3D volumetric assessment might even highlight such improvement more. It was previously demonstrated that LV-GLS was incremental to LVEF and WMSI to predict LV functional recovery and clinical outcome after STEMI [33, 34]. In a study comprising 1041 STEMI patients, 2D-GLS > -15% (median-derived cutoff point) was independently associated with 2D-measured LV dilatation at follow-up [35]. Whether or not the same 2D-GLS cutoff point might have predictive power to anticipate changes in LV 3D-volumes at follow-up needs to be further elucidated in larger-scale studies.

In line with LV volumetric differences among our study groups, follow-up SI was significantly higher among the adverse remodeling group compared to the reverse remodeling one. There was a tendency towards increased LV sphericity among those with minimal change compared to the reverse remodeling group, however statistically non-significant. Mannaerts et al. demonstrated that 3D-derived sphericity index > 0.25 was an earlier and more accurate predictor of remodeling following acute MI than other clinical, electrocardiographic or echocardiographic variables [26]. However, SI by itself is subject to changes in LV EDV and long-axis measurements, reflecting a definite association rather than being a predictor of remodeling.

Results of our study demonstrated a cutoff of 15% reduction in ESV defining reverse remodeling to discriminate prognosis. This was in accordance with that previously described by most studies [17]. On the other hand, our results proposed a cutoff of 12% increase in EDV defining adverse remodeling. Accordingly, a substantial number of patients from the minimal geometric change group were subsequently reclassified as having adverse remodeling. Furthermore, a cutoff of − 16.4% for the follow-up GLS was associated with MACE. Proper structural and functional assessment of patients post-STEMI, identifying those at risk of adverse remodeling has an important therapeutic implication. Treatment goals should be set at attenuating the adverse remodeling process as well as enhancing reverse remodeling. This can be achieved by directing intensified treatment of such patients starting from the early acute and post-acute phase of the infarction and thereafter.

## Limitations

The relatively small sample size was among the limitations of our study. There was no fixed time point for the in-hospital baseline echocardiographic examination that ranged from 24 to 72 h, however, this was the case with most studies with no consensus as to the proper timing of echocardiographic examination post primary PCI. The use of 3D-GLS (still regarded experimental and not available with every software package) might have been even more accurate in relation to 3D LV volumes and remodeling indices. However, 2D-GLS images were acquired in the same setting as the 3D-volumetric images, using the same vendor, and were analyzed later offline with the same software. Larger sample size with more focus on a discriminatory cutoff point of either 2D- or 3D-GLS, as an early functional predictor of 3D LV geometric changes among STEMI patients undergoing PPCI is recommended.

## Conclusion

Our study identified a considerable proportion of patients who did not achieve reverse remodeling and were at the verge of developing adverse remodeling. Following acute MI, two-dimensional GLS was associated with and potentially predictive of changes in LV volumes as detected by three-dimensional echocardiography. This might have therapeutic implications to abort adverse remodeling and enhance reverse remodeling.

## Data Availability

All data generated or analyzed during this study are included in this article.
