# Correction to: Three-dimensional echocardiographic assessment of left ventricular geometric changes following acute myocardial infarction

**DOI:** 10.1007/s10554-023-02855-5

**Published:** 2023-07-17

**Authors:** Heba M. El-Naggar, Alaa S. Osman, Mohamed A. Ahmed, Amr A. Youssef, Tarek A. N. Ahmed

**Affiliations:** Department of Cardiovascular Medicine, Assiut University Heart Hospital, Assiut, Egypt 71526

In the original publication of the Fig. 2 there is a typo error incorrectly published. The corrected figure is given in this Correction. The original article has been corrected.

The original article has been corrected.

